# Gross’s Pathological Anatomy

**Published:** 1845-12

**Authors:** 


GROSS'S PATHOLOGICAL ANATOMY—*Second Edition.*

We have just received from the publishers, Barrington & Haswell, Philadelphia, and James Maxwell, Louisville, a copy of the second edition of this comprehensive and highly valuable work. Of the first, we were (in several numbers of this Journal) enabled to speak in terms of decided commendation; but the second, thoroughly revised and greatly enlarged, has still stronger claims on our applause. It is comprised in a royal octavo volume of more than 800 pages, on fine paper and in beautiful letter press; and, besides about 250 wood cuts, has many faithfully colored engravings.

Intending, in our next number, to give a more extended notice, we shall limit ourselves, at present, to the mention of some of the new matters introduced into this edition. Among these, we observe, as distinct chapters or sections, *Fistules*, *Pneumatosis*, *Polypes*, *Colloid*, *Softening of the Brain*, *Tubercular Arachnitis*, *Internal Strangulation*, *Typhoid Fe-*

*ver, and Wounds of the Intestines;* on the last of which our author, since his first edition, has made an extensive and varied series of experiments, which were published in this Journal, soon after they were completed. Several other articles, as tubercle, suppuration, &c., appear to have been re-written, and, generally, the work affords evidence of careful revision, and many valuable additions.

As this is the first and only systematic work on Pathological Anatomy, which America has yet produced, the profession in the West may justly feel proud, of its second and improved appearance among them. To them, indeed, it must prove a work of greater value than any from abroad, as it contains so many observations on the pathology of the diseases which they are daily called upon to treat. That it will henceforth be regarded as a standard work, and introduced into the library of every physician of the interior, who aims either to educate his pupils thoroughly, or keep himself informed on the improvements of this essential branch of medical and surgical science, we cannot doubt.

